# Agreement between administrative data and the Resident Assessment Instrument Minimum Dataset (RAI-MDS) for medication use in long-term care facilities: a population-based study

**DOI:** 10.1186/s12877-015-0023-2

**Published:** 2015-03-11

**Authors:** Lisa M Lix, Lin Yan, David Blackburn, Nianping Hu, Verena Schneider-Lindner, Yvonne Shevchuk, Gary F Teare

**Affiliations:** University of Manitoba, Winnipeg, MB Canada; University of Saskatchewan, Saskatoon, SK Canada; Health Quality Council, Saskatoon, SK Canada; Medical Faculty Mannheim, Heidelberg University, Heidelberg, Germany

**Keywords:** Concordance, Long-term care, Medication system, Electronic database, Mental health

## Abstract

**Background:**

Prescription medication use, which is common among long-term care facility (LTCF) residents, is routinely used to describe quality of care and predict health outcomes. Data sources that capture medication information, which include surveys, medical charts, administrative health databases, and clinical assessment records, may not collect concordant information, which can result in comparable prevalence and effect size estimates. The purpose of this research was to estimate agreement between two population-based electronic data sources for measuring use of several medication classes among LTCF residents: outpatient prescription drug administrative data and the Resident Assessment Instrument Minimum Data Set (RAI-MDS) Version 2.0.

**Methods:**

Prescription drug and RAI-MDS data from the province of Saskatchewan, Canada (population 1.1 million) were linked for 2010/11 in this cross-sectional study. Agreement for anti-psychotic, anti-depressant, and anti-anxiety/hypnotic medication classes was examined using prevalence estimates, Cohen’s κ, and positive and negative agreement. Mixed-effects logistic regression models tested resident and facility characteristics associated with disagreement.

**Results:**

The cohort was comprised of 8,866 LTCF residents. In the RAI-MDS data, prevalence of anti-psychotics was 35.7%, while for anti-depressants it was 37.9% and for hypnotics it was 27.1%. Prevalence was similar in prescription drug data for anti-psychotics and anti-depressants, but lower for hypnotics (18.0%). Cohen’s κ ranged from 0.39 to 0.85 and was highest for the first two medication classes. Diagnosis of a mood disorder and facility affiliation was associated with disagreement for hypnotics.

**Conclusions:**

Agreement between prescription drug administrative data and RAI-MDS assessment data was influenced by the type of medication class, as well as selected patient and facility characteristics. Researchers should carefully consider the purpose of their study, whether it is to capture medication that are dispensed or medications that are currently used by residents, when selecting a data source for research on LTCF populations.

**Electronic supplementary material:**

The online version of this article (doi:10.1186/s12877-015-0023-2) contains supplementary material, which is available to authorized users.

## Background

Accurate measures of prescription medication use are important for understanding the health and healthcare use of long-term care facility (LTCF) residents. A large percentage of LTCF residents have complex healthcare needs for which one or more medications may be prescribed [1] and there is potential for inappropriate prescribing in this vulnerable population [2]. Measures of medication use in previous studies about the quality of care and health outcomes of LTCF residents include anti-psychotic use in the absence of a psychotic disorder or related condition, anti-depressant therapy in residents without depression, and repeated use of hypnotic medications [3].

Data sources that capture medication information for LTCF residents include surveys, medical charts, administrative health databases, and patient clinical assessment records. Resident surveys are sensitive to self-report or recall biases, while medical chart reviews are time- and labour-intensive to conduct, particularly for multiple facilities. Population-based sources, including administrative health databases and assessment data like the Resident Assessment Instrument Minimum Data Set (RAI-MDS) [4,5], are ideal for making cross-facility or inter-jurisdictional comparisons [6]. However, these data sources were not originally intended for developing facility-specific or jurisdiction-wide quality of care indicators or for studying health outcomes. Administrative data were developed for health system management and monitoring, while the RAI-MDS clinical assessment data were developed to improve resident care. Administrative data do not contain the rich clinical and functional information that is available in clinical assessments, but assessment data contains less information about disease diagnoses, which are essential to characterize patient risk, than administrative data. Thus, there are advantages and disadvantages to both data sources. Accordingly, studies about the comparability of the data in administrative and clinical assessment data, including data on medication use, are important to aid decisions about which source to use in studies about LTCF residents.

Given this context, the purpose of this study was to estimate agreement between medication information recorded in population-based RAI-MDS and prescription drug administrative data. This agreement analysis was conducted for three medication classes that are frequently prescribed for LTCF residents: anti-psychotic, anti-depressant, and anti-anxiety/hypnotic medications.

## Methods

### Data sources

Data for this study were from the province of Saskatchewan, Canada, which has a population of approximately 1.1 million according to the 2011 Statistics Canada Census, and which has a universal healthcare program and therefore captures virtually all healthcare contacts for the entire population. The province maintains comprehensive health care databases in electronic format and these can be anonymously linked via a unique personal health number [7].

RAI-MDS Version 2.0, prescription drug, and person registry system records from the 2010/11 fiscal year (a fiscal year extends from April 1 to March 31), the most current year available at the time of the study, were used to conduct the research. The RAI-MDS, originally developed in the US by the Centers for Medicare and Medicaid Services, captures information about care and functioning of LTCF residents. This information is collected by trained assessors, usually nurses, who use interviews with the person and family members, consultation with other clinicians, and chart review, to complete a form. Forms are required to be completed within 14 days of LTCF admission, quarterly and annually thereafter, and whenever there is a major change in a resident’s health status. The RAI-MDS also captures dates of LTCF admission and discharge and some characteristics of the LTCF itself. Saskatchewan was the first Canadian province to make the RAI-MDS mandatory in all LTCFs; this requirement was introduced in April 2001, although full implementation was not achieved until 2004.

The prescription drug database contains records of outpatient drugs dispensed to provincial residents eligible for insurance coverage. It does not capture inpatient medications, medications for residents of a small number of LTCFs with in-house pharmacies, and approximately 10% of the population who are covered by a federally funded pharmacare program (e.g., military, federal police, federal prisoners, First Nations residents). Each available record includes the date of dispensation and national drug identification number (DIN). DINs are linked to codes in the American Hospital Formulary System (AHFS) Pharmacologic-Therapeutic Classification System (www.ashp.org), which is used to group drugs with similar pharmacologic, therapeutic, and/or chemical characteristics.

The person registry system captures dates of health insurance coverage, demographic information, and location of residence. The accuracy and completeness of Saskatchewan’s administrative health data for research has been well documented [8-12] although not specifically for LTCF populations. Ethics approval for database access was received from the University of Saskatchewan Biomedical Research Ethics Board. Data were accessed and analyzed at the provincial Health Quality Council in accordance with a standing data sharing agreement between that organization and the Ministry of Health.

### Study cohort

The study cohort inclusion criteria were: (a) resident in a LTCF for at least 60 days, (b) at least one admission, quarterly, or annual RAI-MDS assessment during the residency period, and (c) at least one record for a prescription drug during the residency period. The latter criterion was used to ensure that the cohort included individuals eligible to receive prescription drug benefits.

The assessment date of the first admission, quarterly, or annual RAI-MDS assessment in 2010/11 was the study index date. The observation period extended 30 days before and 30 days after the index date. LTCF residents who did not have continuous health insurance coverage and were not eligible for prescription drug benefits during the study observation period were excluded.

### Study variables

Medication information was extracted from both the RAI-MDS and prescription drug databases. Socio-demographic variables were defined using the person registry data. Additional information on resident and facility characteristics were obtained from the RAI-MDS data.

The RAI-MDS captures the number of days of medication use in the seven-day period prior to the assessment reference date. The following medication classes are included in Section O of the assessment form: anti-psychotic, anti-depressant, anti-anxiety, hypnotic, and diuretic. Based on previous research, anti-anxiety and hypnotic medication classes were combined into a single category because they often have a similar indication and diuretics were excluded because they tend to have lower prevalence [13]. In the prescription drug administrative data, anti-psychotic, anti-depressant, and anti-anxiety/hypnotic medications were identified using generic drug names (see Additional file 1) and AHFS codes from the provincial formulary (i.e., drugs covered by prescription drug benefits). The prescription drug database was searched for dispensations of the three medications in the study observation window. We selected a 60-day observation window because the medications under investigation are commonly dispensed in one-month quantities [14]. A sensitivity analysis was conducted using a 28-day observation window (i.e., 14 days before and 14 days after the index date).

LTCF residents were described on the socio-demographic characteristics of age, sex, region of residence, and income quintile. All measures were defined at the index date. Urban or rural region of residence was assigned based on the postal code contained in the person registry. Urban residents were those living in one of the two health regions in the province that contain major urban centres, while rural residents were those individuals living in the remaining 11 health regions. Income quintile was an area-level measure assigned based on average household income from the Statistics Canada Census. Each individual’s postal code from the person registry was assigned to a dissemination area (DA), the smallest geographic unit for which Census data are reported. The entire Saskatchewan population was then divided into five approximately equal groups according to the DA average household income [15]. Preliminary analysis of the data showed that for three quarters (i.e., 74.0%) of cohort members, the index date corresponded to the initial assessment; accordingly, the resident’s postal code would correspond to the place of resident prior to admission.

Selected chronic diseases identified in the RAI-MDS were also captured for each member of the study cohort, including Alzheimer’s disease, dementia, mood disorders (i.e., anxiety, depression, bipolar), and schizophrenia, which are potential indications for the medications under investigations. These conditions were defined at the study index date using admission and annual assessments, or the most recent admission or annual assessment if the index date was the date of a quarterly assessment because not all diagnoses (e.g., schizophrenia) were captured in quarterly assessments.

Facility characteristics captured in the RAI-MDS data in Saskatchewan include the type of facility and its affiliation. These characteristics were defined at the study index date. LTCFs were classified as special care home or other; the former are public facilities for which residence is determined based on need, while the latter are typically private facilities. LTCF affiliation includes amalgamate, affiliate, and contract. Health regions may operate facilities on their own (amalgamate), or the facility may be operated by an independent health care organisation (affiliate), or through a contract for services with an independent organization (contract).

### Statistical analysis

The study cohort was described using frequencies, percentages, means, and standard deviations (SDs). Crude prevalence estimates (percentages) were calculated for each type of prescription medication. Cohen’s κ was used to estimate agreement between the RAI-MDS and administrative data; 95% confidence intervals (CIs) were also computed. The interpretation of κ adopted in this study was [16]: κ < 0.20 is poor agreement, 0.20 ≤ κ ≤ 0.39 is fair agreement, 0.40 ≤ κ ≤ 0.59 is moderate agreement, 0.60 ≤ κ ≤ 0.79 is good agreement, and κ ≥ 0.80 is very good agreement.

Mixed-effects multiple logistic regression models were fit separately to the data for each medication to test resident and facility variables associated with disagreement between the two data sources [17]. All individuals who were identified as medication users in one data source but not in the other data source were included in the disagreement category, while individuals who were either identified as medication users or as medication non-users in both data sources were included in the agreement category. Two models were fit to the data: (a) null model, which contained a random facility intercept only, to account for clustering of patients within facilities, and (b) full model which contained a random intercept as well as resident and facility fixed-effect covariates. Facility type was excluded because it was collinear with affiliation. The intra-class correlation (ICC) was computed for the null and full models [17] using a latent variable method [18]. The Akaike Information Criterion (AIC) was used to compare model fit between the null and full models [19]. The *c*-statistic, which is equal to the area under the receiver operating characteristic (ROC) curve for dichotomous outcomes, was used to assess discriminative performance [20]; it was estimated for the full model when the clustering effect.

All regression coefficients were exponentiated and reported as odds ratios (ORs), along with 95% CIs. Analyses were conducted using SAS software, version 9.2 [21].

## Results

A total of 9,913 individuals were identified as residents in a Saskatchewan LTCF for at least 60 days in 2010/11. Of this number, 350 (3.5%) did not have an RAI-MDS admission, annual, or quarterly assessment, 477 (4.8%) residents did not meet health insurance coverage or prescription drug benefit requirements and 220 residents (2.2%) did not have at least one prescription drug record during the observation period, leaving 8,866 LTCF residents in the study cohort.

The average age of the cohort members was 84.1 years (SD = 11.4; median = 86.0) and they had resided in a LTCF for an average of 912.0 days (SD = 1381.5; median = 465). More than two-thirds of the cohort was female (68.2%; Table 1). Fewer individuals were in the highest (13.8%) than in the lowest (22.2%) income quintile. More than half (51.4%) of the study cohort had a diagnosis of Alzheimer’s disease or dementia.

**Table 1:** **Characteristics of long-term care facility residents in the study cohort (** ***N*** **= 8,866)**

**Variable**	**Frequency (%)**
**Age Group**	
<65 years	608 (6.9)
65 – 74 years	768 (8.7)
75 – 84 years	2452 (27.7)
85+ years	5038 (56.8)
**Sex**	
Male	2819 (31.8)
Female	6047 (68.2)
**Income Quintile**	
Q1 (Lowest)	1970 (22.2)
Q2	1850 (20.9)
Q3	2146 (24.2)
Q4	1622 (18.3)
Q5 (Highest)	1220 (13.8)
Missing Quintile	58 (0.7)
**Residence Location**	
Rural	4348 (49.0)
Urban	4398 (49.6)
Missing Location	120 (1.4)

A total of 163 facilities were identified in the data; the majority were amalgamates and special care homes. The average number of study cohort members in each LTCF was 54.4 (SD = 53.5; median = 37.0).

**Figure 1:**
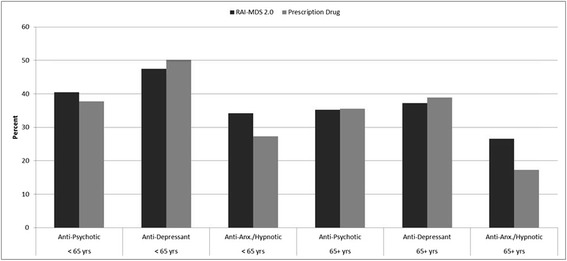
**Prevalence of medication use by type of medication class and age group.**

Crude medication prevalence estimates were 35.7%, 37.9%, and 27.1% for anti-psychotic, anti-depressant, and anti-anxiety/hypnotic medications, respectively, from the RAI-MDS data. The corresponding estimates for the prescription drug data were 35.8%, 39.6%, and 18.0%. Figure 1 contains crude estimates by age group. Prevalence was highest for anti-depressants in residents less than 65 years of age; the estimate was 47.5% in the RAI-MDS data and 50.2% in the prescription drug data.

Overall estimates of Cohen’s κ indicated very good agreement for anti-psychotic and anti-depressant medications, but only moderate agreement for anti-anxiety/hypnotic medications (Table 2). Estimates were similar for younger and older age groups except for anti-anxiety medications, which had slightly higher agreement in younger than older individuals. Estimates were also similar for males and females, although agreement was slightly higher for females than males on anti-depressant medications. In terms of disagreement between the two data sources, the highest frequency was for residents with anti-anxiety/hypnotics identified in the RAI-MDS data but not in the prescription drug data. For the other two types of medications, there were similar percentages of disagreement between the two data sources.

**Table 2:** **Cohen’s** κ **and frequency (%) of agreement and disagreement of RAI-MDS and prescription drug data on medication class use**

	**Anti-Psychotic**	**Anti-Depressant**	**Anti-Anxiety or Hypnotic**
**Cohen’s** κ **(95% Confidence Interval)**			
All ages	0.85 (0.83, 0.86)	0.83 (0.82, 0.85)	0.40 (0.38, 0.42)
<65 years	0.81 (0.77, 0.86)	0.84 (0.79, 0.88)	0.46 (0.39, 0.54)
65+ years	0.85 (0.84, 0.86)	0.83 (0.82, 0.85)	0.39 (0.37, 0.41)
Males	0.82 (0.80, 0.84)	0.79 (0.76, 0.81)	0.39 (0.35, 0.43)
Females	0.86 (0.84, 0.87)	0.85 (0.84, 0.87)	0.40 (0.38, 0.43)
**Frequency (%) of Agreement/Disagreement**			
Positive Agreement: In RAI-MDS and Prescription Drug Data	2851 (32.2)	3087 (34.8)	1054 (11.9)
Negative Agreement: In neither RAI-MDS nor Prescription Drug Data	5385 (60.7)	5079 (57.3)	5925 (66.8)
Disagreement: In RAI-MDS & Not in Prescription Drug Data	311 (3.5)	273 (3.1)	1347 (15.2)
Disagreement: Not in RAI-MDS & in Prescription Drug Data	319 (3.6)	427 (4.8)	540 (6.1)
**Total**	**8866 (100.0)**	**8866 (100.0)**	**8866 (100.0)**

Given the lower levels of agreement for anti-anxiety/hypnotic medications, we focused only on this medication when modeling the factors associated with disagreement (Table 3). The facility-level variance was significantly different from zero in the null model (variance = 0.05; standard deviation = 0.02), but not in the full model. At the same time, the ICC remained constant at 0.01 for both the null and full models, providing little evidence of a clustering effect in the data. The AIC revealed that the full model (9010.3) was a better fit to the data than the null model (9172.9). Discriminative performance of the full model was moderate (*c*-statistic = 0.55). The ORs for disagreement between the prescription drug administrative data and the RAI-MDS data (Table 3) revealed that the odds of disagreement were higher for residents with a mood disorder and for residents of amalgamate than contract facilities.

**Table 3:** **Odds ratios (ORs) and 95% confidence intervals (95% CIs) and model characteristics for disagreement between RAI-MDS and prescription drug data**

**Variable**	**OR (95% CI)**
85+ years	0.90 (0.72, 1.12)
75 – 84 years	0.97 (0.77, 1.21)
65 – 74 years	0.94 (0.72, 1.22)
<65 years	Reference
Males	0.94 (0.84, 1.06)
Females	Reference
Alzheimer’s/dementia: present	0.94 (0.84, 1.04)
Alzheimer’s/dementia: absent	Reference
Mood disorders: present	1.37 (1.23, 1.54)^*^
Mood disorders: absent	Reference
Schizophrenia: present	1.23 (0.91, 1.65)
Schizophrenia: absent	Reference
Income Quintile: Q1 (lowest)	0.95 (0.79, 1.15)
Income Quintile: Q2	1.03 (0.85, 1.24)
Income Quintile: Q3	1.08 (0.90, 1.29)
Income Quintile: Q4	1.04 (0.86, 1.26)
Income Quintile: Q5 (highest)	Reference
Rural residence	0.93 (0.81, 1.07)
Urban residence	Reference
Affiliate facility	1.22 (0.92, 1.62)
Amalgamate facility	1.33 (1.01, 1.77)*
Contract facility	Reference
**Clustering Effect & Model Discrimination**	**Estimate**
Facility-level variance	0.04 (0.02)
Intra-class correlation (ICC)	0.01
c-statistic	0.55

^*^Indicates an OR that is statistically significant at α = .05.

The sensitivity analysis conducted using the 28-day observation window, produced similar results. Specifically, Cohen’s κ ranged from 0.34 for anti-anxiety/hypnotic medications to 0.78 for anti-psychotic medications.

## Discussion

Deterministic record linkage techniques were used to compare LTCF resident medication information captured in prescription drug administrative data and RAI-MDS data for the entire LTCF population of a Canadian province. Agreement ranged from moderate to very good for three medication classes captured in RAI-MDS data, and was lowest for anti-anxiety/hypnotic medications. For this latter medication class, a diagnosis of a mood disorder and the regional affiliation of the facility were associated with disagreement, although the discriminative performance of resident and facility characteristics was modest.

When compared to prescription drug administrative data, which captures information on medication dispensations, it appears that the RAI-MDS clinical assessment data, which captures information on utilization of medications, contain comparable information for anti-psychotic and anti-depressant medication classes [22]. The small size of the ICC for all medications indicates that there was little variation across facilities in the lack of capture of medication class information in either data source. However, for anti-anxiety and hypnotic medication classes, it appears that there may be some differences across facility affiliations, with those being operated by health regions having slightly higher odds of disagreement compared with those contracted to provide services by the health region. This finding suggests that requirements around completion of the RAI-MDS assessment form may not be the same for different types of facilities. Higher disagreement for residents with mental health conditions may, in part, arise because it is more time consuming to complete the assessment form for residents with complex needs, leading to a higher potential for error or incomplete capture of medication class information.

The high rate of disagreement between the RAI-MDS and prescription drug data for anti-anxiety and hypnotic medication classes may have arisen for a number of reasons that are not captured in the study variables. Not all anti-anxiety or hypnotic medications may be covered by provincial health benefits, although this number will be small [7]. The RAI-MDS assessment form does not contain generic names or DINs, therefore LTCF staff may not be able to correctly identify anti-anxiety or hypnotic medications. A patient prescription might not always be filled through a community pharmacy for anti-anxiety or hypnotic medication classes and therefore might not be captured in the prescription drug database; some medication stocks may be kept in-house in LTCFs if they are used on an infrequent or intermittent basis. As well, for medications used on an infrequent basis, the observation window may need to be substantially widened in order to achieve a high estimate of agreement if consumption does not occur shortly after dispensation. However, we found that moving from a 28-day to 60-day window had almost no impact on prevalence estimates for this type of medication.

There are a number of considerations when choosing between prescription drug administrative data and RAI-MDS data for measuring medication use in residents of LTCFs. Databases that contain prescription drug dispensation records do not capture individuals who are not covered by prescription drug benefit programs; this may represent substantial portions of the population in some jurisdictions. In Canada for example, prescription drug dispensations are not routinely captured in some provincial electronic database, or they are captured only for individuals 65+ years of age [23]. At the same time, some jurisdictions may not mandate collection of RAI-MDS data for all LTCF residents, which will result in less than complete population coverage and affect accuracy and generalizability of prevalence estimates. While administrative data capture all drugs prescribed on an outpatient basis, inpatient medications are not typically captured; residents of LTCFs affiliated with acute care inpatient facilities may be missed. While only a small number of medication classes are captured in Section O of the RAI-MDS form, medications administered in the last seven days are captured; this will include medications prescribed while in hospital for individuals who are transferred from an acute care facility to a LTCF.

This study has some limitations. An external validation of each data source was not conducted, although a chart abstraction study or patient medication review would be difficult to undertake given the large number of facilities and patients that would have to be sampled to produce generalizable results; as well such a study would be very expensive to undertake. The discriminative performance of the model covariates was moderate for all of the medications, suggesting that other covariates should be considered as predictors of disagreement. As well, there may be unmeasured confounding in the model due to the limited set of covariates available for consideration. For example, facility characteristics such as number and type of staff might be useful to consider, but are not currently captured in electronic databases.

## Conclusions

This study of agreement between medication classes captured in prescription drug administrative data and clinical assessment data from the RAI-MDS suggests that the medication information contained in both data sources is consistent for anti-psychotics and anti-depressants. For anti-anxiety and hypnotic medication ascertainment, low agreement between the two data sources was not associated with resident socio-demographic characteristics and was only weakly associated with resident disease diagnoses or facility type. While we hypothesize that agreement may be associated with the pattern or frequency of use in LTCF residents, further research is required to draw conclusions about these effects.

Research about the optimal source of data to measure medication use in LTCF populations is important for a number of reasons. Medication use is a risk factor for adverse health outcomes such as hip fracture. Accordingly, failure to include measures of medication use in prediction models represents a potential source of bias due to unmeasured confounding that could lead to erroneous conclusions [24]. Accurate measurement of medication use is also critical to minimize variations in prescribing practices.

## Additional file

Additional file 1:
**Generic names of investigated medications in the prescription drug data.**
