# Supplementary material for: Agreement between administrative data and the Resident Assessment Instrument Minimum Dataset (RAI-MDS) for medication use in long-term care facilities: a population-based study
Source: BMC Geriatr. 2015 Mar 11;15:24. doi: 10.1186/s12877-015-0023-2 (PMC4359405; doi:10.1186/s12877-015-0023-2)
Supplement: Additional file 1: — Generic names of investigated medications in the prescription drug data. [file 12877_2015_23_MOESM1_ESM.docx]

**Additional Files**

**Additional File 1 - Generic Names of Investigated Medications in the Prescription Drug Data**

| **Anti-Psychotic** | **Anti-Depressant** | **Anti-Anxiety or Hypnotic** |
| --- | --- | --- |
| chlorpromazine | amitriptyline | alprazolam |
| clozapine | bupropion hcl | bromazepam |
| flupenthixol decanoate | citalopram hydrobromide | chlordiazepoxide |
| flupenthixol dihydrochloride | clomipramine hcl | clorazepate dipotassium |
| fluphenazine decanoate | desipramine hcl | diazepam |
| fluphenazine hcl | doxepin hcl | flurazepam hcl |
| haloperidol | duloxetine hydrochloride | lorazepam |
| haloperidol decanoate | fluoxetine | oxazepam |
| loxapine succinate | fluvoxamine maleate | temazepam |
| olanzapine | imipramine | triazolam |
| pericyazine | maprotiline | buspirone |
| perphenazine | mirtazapine | chloral hydrate |
| pimozide | moclobemide | hydroxyzine |
| pipotiazine palmitate | nortriptyline | methotrimeprazine |
| prochlorperazine | paroxetine hcl |  |
| quetiapine | phenelzine so4 |  |
| risperidone | sertraline hydrochloride |  |
| thiothixene | tranylcypromine so4 |  |
| trifluoperazine | trazodone |  |
| ziprasidone | trimipramine |  |
| zuclopenthixol acetate | venlafaxine hcl |  |
| zuclopenthixol decanoate |  |  |
| zuclopenthixol dihydrochloride |  |  |
